# Hospital Meetings, &c.

**Published:** 1898-12-31

**Authors:** 


### HOSPITAL MEETINGS, &C.

### HOSPITAL FOR SICK CHILDREN.

A special court of the governors of the Hospital for Sick Children, Great Ormond Street, was held at the hospital on Wednesday afternoon, 21st instant, to sanction the expenditure of a sum not exceeding £5,000 for altering, adapting, and equipping the nurses' house, and also to approve and confirm the past action of the Committee of Management in commencing the expenditure on this work before the sanction of the court had been obtained. Mr. Arthus Lucas, the chairman of the committee, who presided, gave a brief account of what had been done in the matter of the disposal of the adjoining property purchased last year. The church, he said, had been by agreement removed; the hospital, which was so old as to be almost tumbling to pieces, had been pulled down. The convent, though structurally in good order, was otherwise in such a state as to require considerable attention. It was quite impossible for them to put their nurses into a place which was unventilated, unwarmed, in which there was not a single bath-room, and in which the drainage was of the most primitive character. It was also very dirty, not having been painted or repaired for many years. The committee had to consider how best to make their nurses comfortable, and to that end they had decided to heat the house by means of radiators; to put in seven baths; and to instal a system of fire hydrants, the floors being of wood. Lighting was a serious question, and after much deliberation they had decided to adopt the electric light. Then they had to face the all-important question of drainage, and the time taken to prepare the estimate was the reason the court had not been called earlier. The £5,000 for the expenditure of which sanction was asked would be divided as follows: For drainage and lighting, £2,174; for general work and furnishing, £2,368; leaving £458 for contingencies, the whole of which, however, they hoped they would not have to spend. By a wise rule of the hospital, the committee could not expend a larger sum than £500 without the consent of the Governors, hence that meeting. They already had the money, legacies to the amount of £2,000 having fallen in that year, while a further £3,000 under the same head would come in next year. The committee considered they could not apply legacies better than in such constructional work as had been described. He could assure them there was no extravagance contemplated; they were bound to provide for the comfort and health of their nurses, but there would be no undue luxury. The work would be carried out as economically as possible under the supervision of their own clerk of the works. He wished to remind the governors that in 1900 a further sum of £20,000 would be required for the purchase fund, and he announced that their president, the Duke of Fife, had promised a further £100 in addition to the £100 he had already given.

The requisite motions were duly put and carried without dissent, and the meeting separated.

